# Influence of income on diet quality and daily iron and zinc intake: analysis of the National Diet and Nutrition Survey of British females aged 11–14 and 15–18 years

**DOI:** 10.1007/s00394-022-03000-z

**Published:** 2022-09-23

**Authors:** Michelle Thomas, Lisa Coneyworth, Simon Welham

**Affiliations:** Division of Nutritional Science, University of Nottingham, Sutton Bonington Campus, Sutton Bonington, Leicestershire, LE12 5RD UK

**Keywords:** Adolescent females, Iron, Zinc, Household income, Diet quality index-adolescents

## Abstract

**Purpose:**

A negative socio-economic gradient exists for diet and health outcomes. Since cheaper diets are associated with increased energy and lower nutrient density, we investigated the influence of income on iron and zinc intakes and overall diet quality for adolescent (DQI-A) females aged 11–18 years.

**Methods:**

National Diet and Nutrition Survey (NDNS years 7 and 8) data for iron and zinc intake and overall diet quality was assessed by household income quintile across females aged 11–18 years.

**Results:**

Equivalised household income positively correlated with Diet quality index for adolescents (DQI-A) (*P* < 0.001) Females aged 15–18 years in income quintiles (IQs) I and 2, had a greater proportion of respondents with low to intermediate DQI-A score compared to higher IQs (*P* = 0.002). NDNS data showed intake was negatively influenced by income amongst females aged 11–14 years for iron (*P* = 0.009) and zinc (*P* = 0.001) with those from the lowest incomes consistently consuming significantly less than those from the highest. DQI-A was positively correlated with iron intakes for 11–14 (*P* = 0.001) and 15–18 years (*P* < 0.001). Forty-one percent of 15–18-year-olds plasma ferritin stores were below the 15 µg L^−1^ and 21% had some form of anaemia. Cereal and cereal products were the greatest contributors to iron in all groups.

**Conclusion:**

Females in the lowest income groups are at greater risk of lower overall diet quality and inadequate iron and zinc intakes. Amongst older adolescents, there is evidence of iron stores being depleted and an increased prevalence of anaemia.

**Supplementary Information:**

The online version contains supplementary material available at 10.1007/s00394-022-03000-z.

## Introduction

Iron and zinc are essential dietary minerals fundamental for growth and development [1, 2]. During adolescence, defined as the period spanning 10–19 years, females’ physiological requirements for both minerals are increased due to the onset of puberty, [3] increased growth and energy requirements [4] and loss due to menstruation [5]. This, coupled with low dietary intakes, can result in a low iron and zinc status [6]. During the adolescent years, zinc accumulates in muscle and bone at an increased rate and sub-optimal intakes are associated with poor growth and reduced appetite [4]. However, evidence suggests that their provision remains inadequate for many. The prevalence of anaemia in non-pregnant women in the UK is currently estimated at 14% [7] and levels in adolescent girls have previously ranged between 10 and 20% [8]. Low dietary intakes of both minerals may be influenced by economic status. Children living in a household where the occupation is listed as manual are more likely to have a daily iron intake below the LRNI compared to children living in households where the occupation is managerial or professional [9]. Insufficiency of either mineral may negatively impact adolescent females’ physical and cognitive development [4, 10]. Sub-optimal iron intakes have been found to limit female adolescent cognitive function and school performance, whilst an increase in iron status improved learning [8, 11]. This implies that deficiency may be felt in the economic potential of adulthood [12].

Optimal intakes of either nutrient are required to ensure an effective immune response against invading pathogens and lessen the severity and duration of illness [13–16]. Iron and zinc deficiency has been shown to be a factor in the recurrence of childhood respiratory tract infection whilst zinc supplementation in children decreases the incidence and prevalence of pneumonia [13].

The factors which influence an adolescent female’s dietary mineral intake are many and they include but are not limited to increased autonomy and decision-making around food, including frequency of eating out of home [4], dietary preference such as the adoption of vegetarian or vegan diets [17] as well as the influence of their peers [4]. Furthermore, food available in the school environment is also a factor in the diet of adolescent females and free school meals can be a way to increase the intakes of healthy foods [18] (Table 1).

**Table 1:** Percentage of females aged 11–14 and 15–18 years with “non-plausible” energy intakes and summary description of weight, BMI and food energy intake from the National Diet and Nutrition Survey (NDNS) by income quintile

Females		Total population		Income Quintile										
				1		2		3		4		5		*P* value
Number of “non-plausible” energy (kcal) reporters (%)	Age 11–14	41	(37)	11	(48)	5	(31)	12	(55)	7	(25)	6	(29)	0.156
	Age 15–18	57	(50)	11	(58)	17	(50)	5	(26)	16	(57)	8	(53)	0.252
Weight (kg) (S.E.M)	Age 11–14	52.5	(1.36)	51.3	(3.49)	50.7	(3.77)	52.3	(2.57)	52.6	(2.33)	55.1	(3.52)	0.838
	Age 15–18	64.0	(1.24)	66.8	(3.73)	62.9	(1.97)	57.7	(2.26)	65.5	(2.75)	67.9	(3.37)	0.131
BMI (S.E.M)	Age 11–14	21.1	(0.46)	21.1	(1.04)	20.6	(1.37)	21.6	(0.94)	20.7	(0.87)	21.7	(1.21)	0.631
	Age 15–18	23.8	(0.44)	24.8	(1.32)	24.1	(0.79)	21.1	(0.66)	24.2	(1.01)	24.6	(0.82)	0.052
Total energy diet only (kcal) (S.E.M)	Age 11–14	1165	(41.03)	1096	(86.12)	1238	(144.23)	1047	(80.17)	1321	(55.29)	1283	(42.38)	0.118
	Age 15–18	1225	(35.32)	1207	(83.24)	1224	(74.62)	1189	(85.76)	1250	(68.06)	1223	(87.28)	0.992

Number of respondents with a “non-plausible” total food energy (kcal) intake. Results are shown as mean and S.E.M for Total food energy (kcal) for “non-plausible” reporters. BMI and weight (kg) are shown as mean and S.E.M for the total population with valid weight and equivalised income. Number of participants included in analysis females 11–14 years (*n* = 110), IQ1 *n* = 23, IQ2 *n* = 16, IQ3 *n* = 22, IQ4 *n* = 28, IQ5 *n* = 21. Females 15–18 years (*n* = 115), IQ1 *n* = 19, IQ2 *n* = 34, IQ3 *n* = 19, IQ4 *n* = 28, IQ5 *n* = 15

However, during lockdown it was found children consumed fewer fruit and vegetables especially among poorer groups [19]. It is of concern that socioeconomic status has been shown to be positively associated with micronutrient intake [20] and there is evidence of a social gradient between diet quality and health outcome [21]**.** The ability to provide adolescent females with diets that align with nutritional guidelines is negatively impacted by household income. Diets more closely aligned with the government dietary guidelines may cost up to twice as much as those which are not [21, 22]**.** The cost of food may negatively influence the diversity of the diet and as such reduce the potential for obtaining an optimal quantity of micronutrients in low-income households. The price of food is a significant factor in determining purchasing decisions for low-income groups [21, 22], and cheaper diets are frequently associated with increased energy density and lower nutrient quality compared to higher-cost diets [23].

A recent report from Public Health England which analysed the National Diet and Nutrition Survey (NDNS) for all years found daily iron and zinc intake significantly increased with household income for children aged 1.5–3 and 4–10 years. A trend for increased iron and zinc intake with increasing household income was additionally seen for adolescents aged 11–18 years [24].

If females from poorer households consume lower intakes of iron and zinc and have an overall lower diet quality, then it is important to identify the barriers to obtaining an adequate intake of both minerals. The sources of the minerals in the diet as well as the eating occasions which are contributing to intakes, such as school meals, are required to be known for the development of interventions to reduce their risk of deficiency. In this study, we, therefore, set out to establish the extent to which iron and zinc intakes and overall diet quality amongst adolescent females are affected by household income and identify differences in types of foods consumed and eating occasions which might indicate potential routes for intervention.

## Materials and methods

Data for years 7 and 8 (2014/15–2016/16) of the UK NDNS rolling programme were sourced from the UK Data Service [25]. Years 7&8 were chosen as they comprised the most recent version of the survey at the time of study and provided values of equivalised household income, in addition to indices of multiple deprivations (IMD) as quintiles from 1 ‘least deprived’ to 5 ‘most deprived’.

### Overall diet quality of females aged 11–18 years

NDNS food level data provides details of the type and quantity of food consumed. We used the variables ‘Food name’ and ‘Sub food group description’ to assign food groups to the categories laid out within the diet quality index for adolescent (DQI-A) as per previous studies [26, 27]. The DQI-A is a validated tool comprised of three components; dietary quality (DQ), diet diversity (DD) and dietary equilibrium (DE) and is based on food groups within the Flemish food-based dietary guidelines. These are similar to the UK food-based dietary guidelines. This tool is validated and was used in the HELENA Study which assessed the DQI of Adolescents in 10 European cities [26, 27]. Milk alternatives were placed within the milk and dairy category. Savoury sauces and pickles, nutrition powders, artificial sweeteners and dietary supplements were not included in the analysis.

### Calculation of DQI-A

The diet quality index for adolescents was derived by calculating a mean score of the 3- or 4-day diet diaries for each of the participants for each of the DQI-A components: diet quality, diet diversity and dietary equilibrium and dividing by 3. Foods were allocated to either a preference group (recommended for consumption), an intermediate group or a low nutrient, energy-dense group and assigned a value of 1, 0 or – 1, respectively [26]. The diet quality component is aligned with food-based dietary guidelines and is concerned with making optimal food choices from each of the food groups [26, 27]. Food weighting values are multiplied by the quantity (physical weight) of food consumed. Results are summed and divided by the sum of total food consumed (g), then multiplied by 100. The diet diversity component represents the variety of food groups within the diet and is derived by averaging the total weight of food consumed and applying serving sizes as previously described [27]. A score of 1 is given if weight of food in the 9 recommended food groups equals or exceeds the recommended serving size for that food group, 0 if below the recommended serving size. The diet diversity score is summed, divided by 9 and multiplied by 100. Dietary equilibrium component is calculated by subtracting the results from ‘dietary adequacy’ (which is concerned with meeting minimum recommended intakes) from ‘dietary excess’ subcomponents (which is concerned with exceeding the upper limits of recommended intakes) and multiplying by 100 [26]. The higher the score the better the quality of the diet. Scores for DQI-A range from − 33 to 100. Scores of − 33 to 0 typically indicate a low diet quality, > 0 to 33 intermediate, > 33 to 66 good and > 66 very good [27]. We further condensed the values into two groups for the purpose of Chi-Square analysis. These were − 33 to 33% (low) and 33 to 100% (high).

### Iron and zinc intake of females aged 11–14 and 15–18 years in the UK

Person-level estimated daily average intake of micronutrients for iron and zinc was available for children and adolescents (11–18 years of age). Mean values for iron and zinc were compared with age and gender-specific reference nutrient intakes (RNI) and lower reference nutrient intake (LRNI). Income quintiles (IQ) were created from established equivalised household income data provided by the NDNS for children aged 1–18 years in SPSS (IQ1 < £12,152.43, IQ2 ≥ £12,152.43, IQ3 ≥ 19,230.42, IQ4 ≥ £27,541.95 and IQ5 ≥ £43,402.43). We created a separate variable of daily equivalised household income by dividing equivalised household income by 365 for use in liner regression analysis to understand the relationship between income, iron and zinc intakes and DQI-A. The contribution of food groups to average iron and zinc intakes was calculated from food level data. This was completed for total intakes in addition to separate analyses which examined solely those foods consumed in school. For analysis of school intakes, only foods which comprised either hot food provision or alternative foods purchased on school premises were included.

### Sensitivity analysis “plausible” reporters

“Plausible” reporters of energy intakes determined by calculating the Energy Intake/Basal Metabolic Rate (EI/BMR) and applying physical activity level (PAL) values and cut-off points (age dependent). “Plausible” reporters were participants with EI/BMR ratio within the cut-off point values as previously published [28]. Low reporters were included in the analysis but highlighted to indicate caution in the interpretation of findings.

### Statistical analysis

The statistical analysis was performed using the SPSS Statistical package (Version 26.0 and 27.0. Armonk, NY: IBM Corp, Released 2020). Participant characteristics are presented as means and standard error of the mean (S.E.M). DQI-A results are presented as means ± S.E.M.

Linear regression is used to determine whether daily equivalised household income predicts DQI-A and if equivalised daily household income and DQI-A predict variance in iron and zinc intakes. Chi-square analysis was performed to understand if the representation of participants with low to intermediate (− 33 to 33) and intermediate to high DQI-A (> 33) scores varied across income quintiles and if the representation of “plausible” energy reporters differs across income quintiles. Normality of the distribution of the food data as grouped by the DQI-A tool was evaluated using Shapiro–Wilks. Results of all food groups indicated non-normally distributed data (*P* < 0.05). Non-parametric Mann–Whitney U tests were performed for comparison of the total weight of food consumed within each of the food groups for low to intermediate and intermediate to high DQI-A scorers. Results are presented as median with IQR.

Pearson’s correlation was used to compare DQI-A, dietary mineral intakes with plasma ferritin, haemoglobin, and zinc levels.

The National Diet and Nutrition Survey person-level dietary data were also analysed, with descriptive statistics computed for each of the population groups for the percentage of the population meeting the RNI, percentage of the population with an intake below 90% of the RNI and percentage of the population with intakes below the lower reference nutrient intake (LRNI). Food level data were grouped as per the NDNS results for food groups.

Normality of the data was determined, and the appropriate parametric or non-parametric test conducted. Kruskal–Wallis tests were performed to determine variation between daily iron and zinc intake across different income quintiles. Participants were excluded from the analysis for income quintiles when a value for equivalised household income was not provided.

## Results

### Population characteristics

The NDNS data for years 7 and 8 contained dietary information for 272 females aged 11–18 years but only 231 had details of household income (mean age 14.7 ± 0.15 years), of which 11–14 olds accounted for 47.8% (12.6 ± 0.10 years)- and 15–18-year-olds 52.2% (16.6 ± 0.1 years). The largest proportion of the respondents living in the most deprived areas of the UK was from the lowest income quintile (IQ1; 36.6%), whilst the largest proportion in the least deprived areas were those with the highest income (IQ5; 32.4%). Amongst females aged 11–14, 26.1% of those in IQ1 lived in the most deprived areas of the UK and 27.3% of IQ5 lived in the least deprived areas, whilst for females aged 15–18 years these proportions rose to 47.6% and 40.0%, respectively.

### Overall diet quality

All diet quality assessments varied positively with income and typically the food groups consumed in a greater quantity by those with a higher DQI-score were from the food groups associated with a higher micronutrient composition such as ‘fruits’ (*P* < 0.001), ‘vegetables’ (*P* < 0.001), ‘milk products’ (*P* < 0.001), ‘bread and cereals’ (*P* = 0.002), whilst those associated with a higher energy content such as ‘snacks and candy’ and ‘sugared drink and fruit juice’ were consumed in similar quantities in both low and high DQI-A scorers (*P* = 0.871; *P* = 0.793, respectively). The food groups remained similar when broken down by age group with 11–14-year-olds with a higher DQI-A score consuming a greater weight of food from the food groups listed above and this was mostly the same for 15–18-year-olds.

### Representation of participants with low or high DQI-A between the income quintiles

The DQI-A (ranges from − 33 to 100% [27] was 38.7 ± 0.92 on average across the population. When separated into age categories, DQI-A was 39.3 ± 1.2% and 38.2 ± 1.4% for 11–14-year-olds and 15–18-year-olds, respectively. DQI-A varied considerably from -5.78 up to 72.74 and this range was present in all income quintiles. Chi-Square analysis of the data for all females found that having a low to intermediate (− 33 to 33%) or intermediate to high DQI-A (> 33%) was moderately dependent on the income quintile (Cramer’s *v* = 0.307). A greater proportion of females in IQ1 and IQ2 had a DQI-A score of 33% or below (40.9% and 49.0%, respectively) compared to IQ3 (32.6%), IQ4 (25.0%) and IQ5 (5.4%; *P* < 0.001). This was predominantly driven by outcomes for 15–18-year-olds (*P* = 0.002; Cramer’s *v* = 0.379) as the association was not significant for the 11–14 s (*P* = 0.282). In the older group, the proportions below DQI-A of 33% rose to 47.6% and 55.9% for IQs 1 and 2 (*P* = 0.002).

### Relationship between equivalised household income and diet quality component (DQc) of DQI-A

The dietary quality component of DQI was low for both 11–14 (15.3 ± 2.85%) and 15–18-year-olds (11.6 ± 3.13; Range = − 100 to + 100). Income was directly associated with DQc (*P* = 0.001; β 0.216). For every £1 increase in weekly equivalised household income DQc increased 0.135%. Income was not a predictor of DQc for females 11–14 years (*P* = 0.293) but was for 15–18-year-olds (*P* < 0001) with every £1 increase in income resulting in an increase of DQc of 0.221%.

### DQI-A and weight of food consumed within food groups

For those with a low to intermediate DQI–A score (*n* = 73) their diets predominantly comprised a lower weight of fruits (26.3 g, IQR 105.3 g) compared to intermediate to high DQI-A score (*n* = 158; 112.3 g, IQR 149.4 g; *P* < 0.001). They also consumed fewer ‘vegetables’ (39.1 g, IQR 54.2 g vs 84.3 g, IQR 84 g; *P* < 0.001), ‘milk products’ (75.0 g, IQR 112.2 g vs 156.3 g, IQR 170.1 g; *P* < 0.001), ‘bread and cereal’ (94.3 g, IQR 54.5 g vs 114.6 g IQR 83.5; *P* = 0.002) and ‘fats and oils’ (5.3 g, IQR 9.6 g vs 9.7 g, IQR 10.5 g; *P* = 0.016) compared to intermediate to high DQI-A scorers. The food groups ‘sugared drinks and fruit juice’, ‘snacks and candy’, ‘potatoes and grains’ ‘meat, fish and substitutes were all consumed in similar amounts between the DQI-A groups (*P* = 0.703; *P* = 0.871; *P* = 0.628; *P* = 0.912, respectively). The pattern was similar for both age categories with 11–14 low DQI-A consuming lower quantities of vegetables (44% less), fruits (67% less), ‘meat, fish and substitutes’ (17% less) and milk products (58% less) than high DQI-A and for 15–18-year-olds these values were 59%, 72% and 39% for vegetables, fruit and milk products, respectively (*P* < 0.001). It was additionally of note that 15–18-year-olds in the low DQI-A group consumed 36% more free sugars than those from the higher DQI-A group (*P* = 0.004).

### The influence of household income on iron, zinc and energy intake in UK female adolescents

#### Iron

**Fig. 1:**
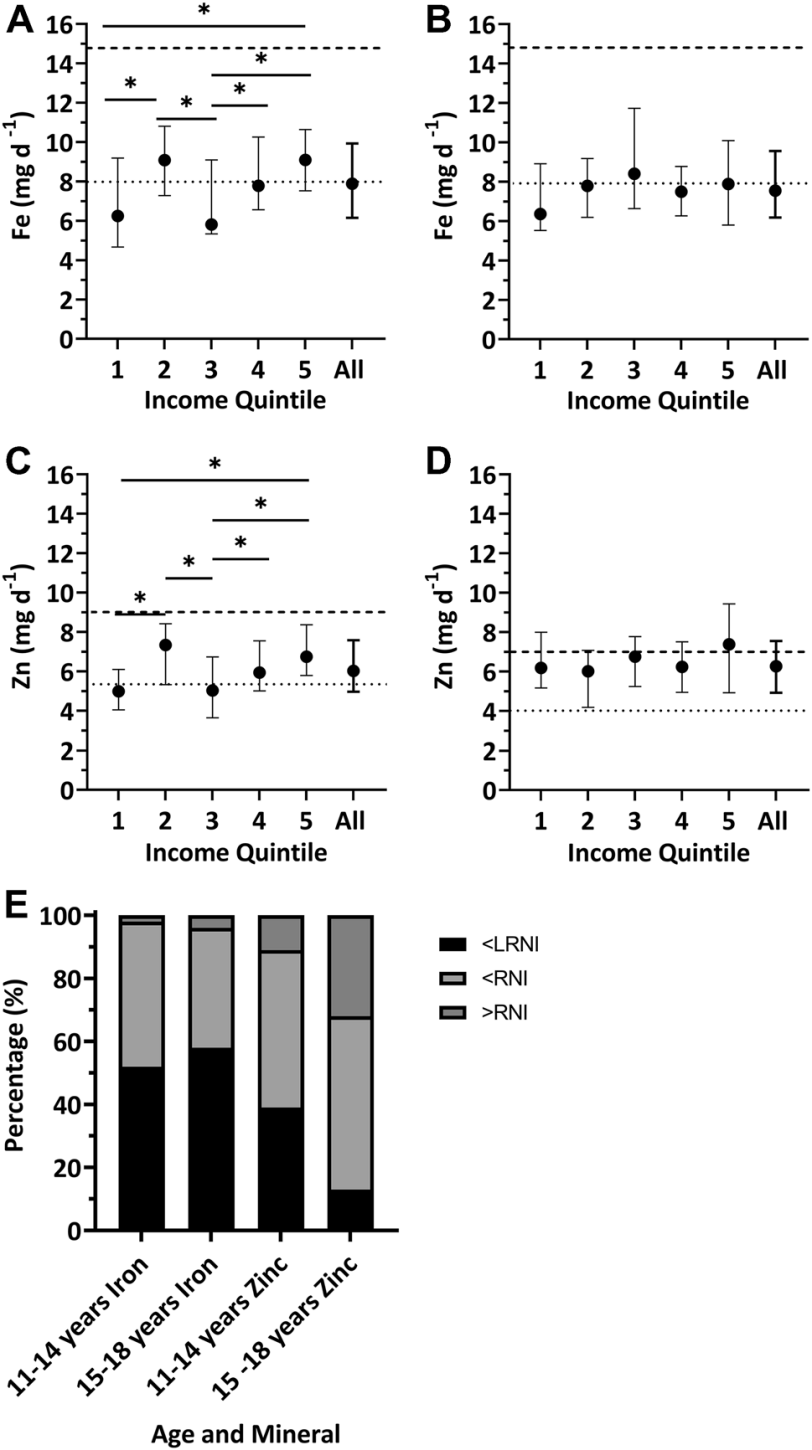
**A**, **B** Median and interquartile range for daily dietary iron intake (mg day^−1^) from food sources only: females aged 11–14 and 15–18 years across income quintiles (IQ). Kruskal—Wallis test was performed in IBM SPSSv26 to evaluate the potential influence of equivalised household income on daily iron intake, post-hoc Mann–Whitney test performed when significance detected at the Kruskal–Wallis stage. Lower bound values for income quintiles are as follows: (IQ1) < £12,152.43, (IQ2) ≥ £12,152.43, (IQ3) ≥ £19,230.42, (IQ4) ≥ £27,541.95, (IQ5) ≥ £43,402.43. Dotted line represents Lower Reference Nutrient Intake (LRNI), dashed line represents Reference Nutrient Intake (RNI). Number of participants included in the analysis with a valid income female 11–14 years IQ1 *n* = 23, IQ2 *n* = 17, IQ3 *n* = 24, IQ4 *n* = 28, IQ5 *n* = 22. Females 15–18 years IQ1 *n* = 21, IQ2 *n* = 34, IQ3 *n* = 19, IQ4 *n* = 28, IQ5 *n* = 15. *Significant at the *P* < 0.05 level. **C**, **D** Median and interquartile ranges for daily dietary zinc intakes (mg day^−1^) from food sources only: females aged 11–14 and 15–18 years across income quintiles (IQ). Kruskal–Wallis test was performed in IBM SPSSv26 to evaluate the potential influence of equivalised household income on daily zinc intake, post-hoc Mann–Whitney test was performed when significance detected at the Kruskal–Wallis stage. Lower bound values for income quintiles are as follows: (IQ1) < £12,152.43, (IQ2) ≥ £12,152.43, (IQ3) ≥ £19,230.42, (IQ4) ≥ £27,541.95, (IQ5) ≥ £43,402.43. Dotted line represents Lower Reference Nutrient Intake (LRNI), dashed line represents Reference Nutrient Intake (RNI). Number of participants included in the analysis with a valid income female 11–14 years IQ1 *n* = 23, IQ2 *n* = 17, IQ3 *n* = 24, IQ4 *n* = 28, IQ5 *n* = 22. Females 15–18 years IQ1 *n* = 21, IQ2 *n* = 34, IQ3 *n* = 19, IQ4 *n* = 28, IQ5 *n* = 15. *Significant at the *P* < 0.05 level. **E** Percentage of females aged 11–14 and 15–18 years with daily iron and zinc intakes below the Lower Reference Nutrient Intake (LRNI) and above or below the Reference Nutrient Intake (RNI). Data sourced from the National Diet Nutrition Survey (NDNS) years 7 & 8 of the rolling programme. Number of participants: females, 11–14 years *n* = 130 and females 15–18 years *n* = 142

Iron intakes of females aged 11–18 years were frequently below the RNI (Fig. 1A and B dashed line). For those between 11 and 14 years (*n* = 130) 98% had an iron intake below the RNI (14.8 mg/day) with 52% being below the LRNI (8.0 mg/day), whilst for females between 15 and 18 years, (*n* = 142) 58%, were below the LRNI, with 96% below the RNI (Fig. 1E).

Daily iron intakes differed significantly across income quintiles (IQ) for females aged 11–14 years (*P* = 0.009) with those in IQ5 (61% of RNI) being significantly higher compared with IQ1 (just 42% of RNI; *P* = 0.014) and IQ3 (39% of RNI; *P* = 0.005). The IQ4 group (53% of RNI) consumed more than IQ3 (*P* = 0.035) and intake in IQ2 was considerably higher than for those in the adjacent quintiles (37% higher than IQ1—*P* = 0.039 and 44% higher than IQ3—*P* = 0.024). Females aged 15–18 showed similar intakes across income quintiles.

Plasma ferritin concentrations were generally in the normal range (41–400 µg L^−1^) but were 27% lower in the 15–18 years group compared with the 11–14 s (*P* = 0.02; Supplementary Table 1). The proportion of 11–14 s who fell below the 15 µg L^−1^ threshold indicator of low iron stores [29] was 10% but amongst the older girls (15–18 years) this reached 41%. Haemoglobin levels exceeded 120 g L^−1^ for the majority, however, 21% of females aged 15–18 years had some form of anaemia, with 14% showing mild (haemoglobin level between 110 and 119 g L^−1^) and 7% moderate anaemia (haemoglobin 80–109 g L^−1^).

DQI-A scores showed a significant positive relationship with iron intakes (β 0.303, *P* < 0.001) with every 1% increase in DQI-A resulting in a 0.066 mg increase in iron for all participants. This was similar for 11–14 (β 0.301, *P* = 0.001; 0. 069 mg increase per 1% DQI-A) and 15–18-year-olds (β 0.306, *P* = 0.001). Neither ferritin nor haemoglobin correlated with DQI-A scores in either group.

#### Zinc

Zinc intakes were low in both age groups (Fig. 1C and D) with 39% of females aged 11–14 years and 13% in the 15–18 years category having a zinc intake below the LRNI (5.3 mg day^−1^; Fig. 1E). Only 11% of 11–14-year-old girls achieved the RNI for zinc (9.0 mg/day), whilst 68% of 15–18 years group were below their respective RNI (7.0 mg/day) (Fig. 1E).

The zinc intakes of females aged 11–14 years also differed with household income (*P* = 0.001; Fig. 1C), with those in quintile 1 being the lowest. This group showed a lower consumption (55% of the RNI) compared with IQ2 (81% of RNI; *P* = 0.026) and IQ5 (75% of RNI; *P* = 0.004). Similar to the findings for iron intake, 11–14-year-old females in IQ2 consumed significantly more zinc than those in the adjacent quintiles (32% higher than IQ1—*P* = 0.026 and 40% higher than IQ3—*P* = 0.026). Zinc intake did not differ with income quintile in the 15–18 years group (Fig. 1D).

Daily zinc intakes were positively associated with DQI-A in all (β 0.373, *P* < 0.001), with 0.061 mg (β 0.390 *P* < 0.001) and 0.071 mg (β 0.306 *P* < 0.001) increases for each 1% increase in DQI_A (11–14 and 15–18, respectively).

#### Energy intakes

Females aged 11–18 years with values for body weight and equivalised household income were included in the analysis (*n* = 225) to identify “plausible” and “non-plausible” reporters of energy intakes (kcal). In total, 43.6% of females did not have a “plausible” energy intake. When analysed by age range, 37.3% of 11–14-year-olds (*n* = 110) and 50% of 15–18-year-olds (*n* = 115) did not have “plausible” energy intakes. There were no differences in reporting reliability across income quintiles for either age group (*P* = 0.156, *P* = 0.252, respectively).

### Contribution of different foods to iron and zinc intake

Foods that had the greatest contribution to daily iron intakes were cereal and meat based (hereafter referred to as cereal and meat products; Supplementary tables 2 and 3), with meat contributing an increasing proportion in older groups (*P* < 0.001; Supplementary tables 5 and 6). These, in addition to vegetables, vegetable products and potatoes (hereafter vegetable products) and milk products were significant contributors to zinc intakes.

### Females aged 11–14 years

#### Iron

Most of the iron intake in females aged 11–14 years was from cereal (52%), meat (14%) and vegetable products (12%; Supplementary table 2). Flour-containing foods contributed ~ 35% of the total iron intake whilst breakfast cereals, consumed by 62% of participants, contributed 16%. Although neither the quantity nor proportion of daily iron intake from breakfast cereals differed across income quintiles (*P* = 0.077 and *P* = 0.699, respectively) the total quantity of cereal-based products consumed did (*P* = 0.001; Supplementary Table 4). Of note, females in IQ2 consumed more than those in IQ1 (*P* = 0.047) and IQ3 (*P* = 0.001). Meat products were consumed by 98% of respondents and no differences in intake were observed between quintiles for either meat or vegetables. We estimated the bioavailable iron from each participant’s diet by assuming that the absorption of iron from vegetable sources would be 10% of intake and that from animal sources (all assumed to be haem iron—meat and fish) would be 25% [30]. For those who met the 1.4 mg day^−1^ threshold indicated as necessary for females of 11–18 years old [31], the iron derived from meat and fish was approximately 30% higher than for those who fell short of this level (*P* = 0.026).

#### Zinc

Meat (31%) and cereal products (31%) were the main contributors to zinc intake with milk products providing most of the remainder (16%; supplementary table 3). The percentage contributions of food groups did not vary greatly between those achieving the 9 mg RNI, however, when individuals were separated according to those who achieved 7 mg (the RNI for all older age groups) and those who did not, then milk was shown to provide a significantly higher proportion of zinc (32% higher; *P* = 0.013) than for those below the 7 mg threshold.

### Females aged 15–18 years

#### Iron

Iron in 15–18-year-old females was again predominantly derived from cereal (46%), meat (17%) and vegetable products (15%; Supplementary table 5). All participants reported consuming some form of meat. Again, 35% of daily iron intake was contributed by flour-containing foods. Just 50% reported eating breakfast cereals. This resulted in only 12% iron provision by breakfast cereals. Iron provision from meat and fish combined was similar between those achieving the predicted 1.4 mg day^−1^ threshold compared with those below this level (*P* = 0.485). The proportion of iron obtained from meat was 23% greater than for the 11–14 age group (*P* < 0.001).

#### Zinc

The largest contributor to zinc intake in 15–18-year-old females was meat (35%; Supplementary table 6). Although this did not differ overall by income, the quantity of zinc derived from burgers and kebabs did, being significantly negatively associated with income level (*P* = 0.026). Cereals, milk and vegetables are provided between 11% and 18% each. Vegetable consumption was positively associated with income (*P* = 0.028). Those who consumed less than the 7 mg RNI, obtained a significantly greater proportion (18% higher; *P* = 0.029) of their zinc intake from cereal products compared with those whose intakes exceeded 7 mg.

### Contribution of school foods to iron and zinc intakes

For many, particularly those on low incomes, school food provision would potentially contribute greatly to dietary intake of critical nutrients. We, therefore, determined the intake of iron and zinc from school-provided meals for 11–18-year-olds. Of the respondents who recorded diet diary days during school time, we found that across all ages, 45% consumed school-provided meals of which 78% were cooked. The proportions of children consuming school meals were similar across income groups. Half of the girls who consumed school meals obtained around 25% (26.2% of total; IQR 18.4–35.3%) of their daily iron intakes from them, while for zinc, this was slightly higher at 30.2% of total intake (IQR 24.3–43.9%). School meals should provide 35% of the requirements [32] and we found that this was the case for just 17% and 20% of girls for iron and zinc, respectively across all age groups.

### Impact of education and gender of main food provider

Whilst higher levels of education are usually associated with higher household income and better diets, we found no evidence of a difference in the iron and zinc intakes of females 11–14 (*P* = 0.788, *P* = 0.487, respectively) and 15–18 years (*P* = 0.962, *P* = 0.872, respectively) when living in a household where the main food provider had a degree (*n* = 38, *n* = 32, respectively) compared to those who did not (*n* = 74, *n* = 76, respectively). Gender of the main food provider also was not associated with iron and zinc intakes in both age groups (11–14 years iron *P* = 0.397, zinc *P* = 0.460; 15–18 years iron *P* = 0.164, zinc *P* = 0.413).

### Household income source

Very few respondents were solely dependent on benefits (*n* = 20), whilst there was a number who received benefits in addition to income from employment (*n* = 171). Because of the low numbers of benefits only, both age groups were combined. Whilst females living in a household with income from employment had a numerically greater iron intake (8.23 ± 024 mg day^−1^) compared to females living in a household with income solely from non-working sources (7.78 ± 0.58 mg day^−1^) this was not significant (*P* = 0.539) and this was similar for zinc (employment 6.29 mg ± 0.17 mg day^−1^, solely benefits 5.75 mg ± 0.36 mg day^−1^; *P* = 0.289).

## Discussion

Iron and zinc deficiency continues to be of concern for many children in the UK. Our data indicated a decrease in iron and zinc from food sources amongst females aged 11–18 years compared with observations from previous years particularly amongst the older females [23]. We found, similar to previous work, [23] that income influenced iron and zinc intake with those in the lowest income quintile most frequently consuming the least. We also showed that diet diversity was compromised in those from lower incomes, particularly for older adolescents. These observations suggest that there may be a considerable number of disadvantaged children who not only consume low quantities of iron and zinc but may be further compromised by the composition of the foods that can be afforded.

### Intake levels

Dietary iron and zinc intakes for females aged 11–14 and 15–18 years were low compared to the RNI and for many were below the LRNI, indicating that iron intake was insufficient to meet requirements at a time when the physiological demand to support growth and development is at its greatest [5]. The RNI is set at 14.8 mg day^−1^ for females aged 11–18 years and for non-menopausal women, to account for a typical daily iron loss of 0.8 mg day^−1^, with an additional 0.6 mg day^−1^ due to menstruation, in the face of a bioavailability of iron from food sources of approximately 10% [33]. Therefore, for females to remain iron replete there is a requirement for 1.4 mg of iron to be absorbed from the diet daily [33]. Dietary iron intake for 11–14- and 15–18-year-olds was half of the RNI, indicating suboptimal intakes which, if sustained, could lead to depletion of iron stores and anaemia. We found that 10% of females aged 11–14 years had plasma ferritin levels below 15 µg L^−1^, potentially indicating low iron storage, although this may be more reflective of stores being utilised to support growth and development [5] particularly since haemoglobin levels were normal in this group (Supplementary Table 1). However, a large proportion (41%) of 15–18-year-old females had plasma ferritin levels < 15 µg L^−1^ with 21% of them having haemoglobin levels indicative of anaemia. Sustained suboptimal iron intake and increased physiological requirements may have resulted in the development of anaemia in a subset of the 15–18-year-old girls in this age group. Other factors which may contribute to anaemia, including B12 and folate intake and clinical factors, such as thalassemia, inflammatory conditions and haemolysis were not considered in this study, but they represent far less frequent causes of anaemia than low iron intake. Iron deficiency in the absence of anaemia can have adverse consequences on mental capacity and immune health [34] and importantly, adolescents entering the reproductive years may not have sufficient iron stores to support the increased demand during pregnancy, estimated at 4–6 mg daily [33]. The frequency of anaemia in pregnancy has been recorded at levels as high as 46% in some UK cohorts [35, 36] representing a significant health risk for the mother and developing child [37] and it seems likely that those individuals who have been exposed to moderate iron deficiency during their teenage years, would likely comprise a significant proportion of this anaemic cohort.

The bioavailability of iron differs considerably between animal and plant-based foods. Iron from animal products is more bioavailable as it is in the form of haem iron, of which 25–30% is absorbed via the intestinal haem carrier protein 1 (HCP1 or SLC46A1). Iron from plant-based foods is predominantly in the form of Fe^3+^ which must be reduced to Fe^2+^ to enable its absorption through the divalent metal transporter 1 (DMT1 or SLC11A2). Consequently, only between 1 and 10% of the iron derived from plant sources is absorbed [11]. Zinc and iron are additionally impacted when acquired from plant-based sources, due to the presence of phytic acid which binds divalent ions, thereby inhibiting their absorption [38]. Therefore, diets high in plant material can potentially have a significant negative impact on iron and zinc status even if they contain them in relatively high concentrations. Consumption of antinutritional factors was not analysed in this report, principally due to the dearth of reliable food level data but is a factor which needs be considered in future work to help gain an understanding of the relative impact on status that this may be having in the UK population.

Zinc intake was below the RNI for a large proportion of both age groups (78.3% of all females). We found a significant negative association between intake and household income (Fig. 1), contrary to findings for previous NDNS cohorts [39] which reported no effect. Household inequality has been approximately stable over the last decade but was more volatile prior to 2010 [40], increasing sharply in non-retired households from 2002 to a peak in 2008 just before the economic downturn. The negative effect of declining household income on the ability of families to adequately feed their children is well documented [41–43]. Differences observed between income quintiles for intake in females for both age groups for both iron and zinc, therefore, may reflect a negative impact of early life exposure to inequality. Previous data which did not find an association with household income [39] is derived from individuals who were living through a period of relative stability in the level of inequality (~ 1987–1997). It is of note that children who comprised the 11–14 years cohort in the 2014–2016 NDNS survey would have ranged from 0 to 2 at the start of the steep rise in inequality. It is possible that discrepancies in consumption may link to economic challenges occurring at the very start of their lives.

### Underreporting

Underreporting was widespread and was particularly high for 11–14-year-old females in IQ1 and IQ3 where 48% and 55% had “non-plausible” energy intakes. It has been shown that adolescent females are more likely to underreport energy intakes, particularly those with a higher BMI. Factors such as forgetfulness, eating meals outside of the home and being conscious of body weight and image impact reporting reliability [44] and this is particularly stark for adolescent females as up to 49% of respondents’ energy intakes are low compared to estimated Basal Metabolic Rates (BMR) [45].

The underreporting will have inevitably skewed data in our study to indicate a higher proportion of individuals consuming below the RNI. However, there would remain a significant proportion of girls aged 11–18 years studied who were marginally deficient for iron. This was evident from the numbers of girls aged 15–18 years with haemoglobin levels below the cut-off point for diagnosis of anaemia. Whilst for 11–14-year-olds haemoglobin levels were above the threshold for anaemia, 10% had depleted serum ferritin stores, increasing to 41% in 15–18-year-olds. These values, whilst in themselves are not the best indicators of status, do support the outcomes of low consumption levels seen in the dietary data.

### DQI-A outcomes

The results from our study found DQI-A for females aged 11–18 years overall, was 38.7% indicating average adherence to food-based dietary guidelines. The results for DQI-A in this study are slightly higher compared to a previously published study which reported DQI-A of 31.4% for adolescent females [27]. Overall females in the highest income quintile, DQI-A score was greater than those in the lowest (47.9% compared to 35.1%, respectively) and this was particularly pronounced amongst 15–18 years olds where DQI-A of females with the lowest income quintile was 16 percent lower compared to the females in the highest income quintile. Foods typically thought of as nutrient dense and low energy were consumed in lower quantities among females aged 15–18 years with a DQI-A score below 33% compared to those with a DQI-A above 33%, indicating that diets among girls in this age group in lower income quintiles are worse compared to their higher-income peers. This was supported by the observation that free sugar consumption in those with a low DQI-A was higher than in high DQI-A, and likely a consequence that these girls are making more autonomous dietary decisions.

### Food contributions

The food group which contributed the greatest proportion of dietary iron was cereal products. Of these, the main single contributor was flour (~ 36% for 11–14 years and ~ 34% for 15–18 years). This would suggest that flour contributed ~ 34% of the total iron intake with breakfast cereals providing another 17%. Of the remainder, around 28% was from meat and vegetable products. This highlights the value of appropriate fortification of flour and of consuming breakfast cereals which was not universal in these cohorts. The relative contribution of breakfast cereals to iron intakes suggests that those choosing not to consume them are at significant risk of falling further short of the recommended intake levels. It should also be noted that not all breakfast cereals are fortified equivalently, so there may be some value in standardisation of cereal fortification to help ensure their ability to enable adequate iron intakes.

We noticed a higher contribution (30%) to dietary iron from meat and fish in 11–14-year-old females able to achieve their iron intake requirements compared with those who were not. The widespread consumption of meat across the whole population would suggest that provision of iron from meat sources might represent a viable strategy for increasing iron levels, particularly for those who do not consume breakfast cereals. This may be particularly pertinent for females aged 15–18 years as meat contributed a significantly higher proportion of iron for them than for the younger group. An important barrier to this would be cost. However, meals made from cheaper ingredients, whilst potentially lower in iron concentration, could still provide a cost-effective alternative. Females from lower income quintiles in the 15–18-year age group obtained proportionally more zinc from burgers and kebabs than those from the higher quintiles. A larger proportion of these teenagers may therefore be making their own dietary choices outside of the home than those from wealthier backgrounds. This is likely to impede successful interventions aimed at improving diet quality and diversity as the routes of successful communication will be more limited.

### Food cost

The cost of foods influences the types purchased and diets aligned with government recommendation are more expensive than those which are not [43]. Additionally, food cost is also a factor in the food security of households, especially if available foods are not affordable [46]. Availability and affordability of foods and household food security have recently received attention due to the COVID-19 pandemic which resulted in panic buying of staple foods reducing the availability of lower-cost food items [47]. This reduced the size and quality of the diet of low-income households and increased food insecurity as they do not have the disposable income to purchase foods in bulk or to purchase higher-cost alternatives. During COVID-19 schools were closed and the safety net of school food removed, although families of children eligible for free school meals (FSM) were supported with a £15 voucher per week to provide lunch for their child. However, for many other families on a low income but not entitled to FSM, they had to bear the burden of increased food cost and increased quantities of food to be purchased to cover the meals not provided at school.

When the percentage of the population with an intake below LRNI exceeds 5% it may be a public health concern as clinically relevant deficiencies may occur [48]. This was highlighted in the SACN Iron and Health report [5], which found toddlers, girls and women of reproductive age to be at increased risk of iron deficiency anaemia. This was particularly apparent if they were from low-income groups [5]. Greater provision, therefore, needs to be made for those in low-income groups to support adequate iron and zinc nutrition during childhood with greater emphasis placed on mechanisms which allow the provision of important micronutrients. Novel mechanisms to facilitate access to and consumption of iron and zinc-rich foods in children, particularly those from lower-income households, are required with some urgency. The cost-of-living crisis has seen energy, fuel and food cost all increase in recent times (since late 2021) and disposable incomes decrease. Low-income households experience higher inflation compared to wealthy households [49] and whilst there are government strategies in place to help reduce the burden such as the cost of living support from May 2022 [50] these are one-off payments. The increase in Universal Credit during the COVID-19 pandemic provided households with a steady source of income and the removal of the uplift in October 2021 left many worried they would not be able to feed their families and rely on coping strategies such as reducing the quantity of food consumed and feeding children before adults [51], all of which may have negative impacts on the diet quality and micronutrient intakes of the most vulnerable population groups.

## Conclusion

The overall diet quality of UK female adolescents in the lowest income quintiles is notably worse than for their higher-income peers and this negatively impacts the quantity of iron and zinc consumed. Furthermore, there is evidence for decreasing plasma ferritin and increasing the prevalence of anaemia as females enter their late teen years. Persistent low intakes in the face of high physiological requirements will compound the prevalence of deficiency and adverse health outcomes associated with sub-optimal micronutrient intakes often seen in lower-income groups. Interventions are required to increase iron and zinc intakes in female adolescents across all income quintiles with an emphasis on ensuring diets aligned with government dietary guidelines are accessible and affordable for all to ensure micronutrient intakes are adequate for the avoidance of ‘hidden hunger’ in the lowest income groups in the UK. Notably, we show that increasing income has a direct positive effect on DQI-A which in turn positively impacts iron and zinc intakes. School food is a good vehicle for the promotion of healthy diets and therefore, represents a potential avenue, outside of direct financial support, for improving health outcomes in adulthood and future generations as adolescent females enter the reproductive years.

## Supplementary Information

Below is the link to the electronic supplementary material.

Supplementary file1 Supplementary table 1 Plasma ferritin and haemoglobin levels of females in the UK aged 11–14 and 15–18 years and by income quintile. Supplementary table 2. Percentage contribution of food and food groups to daily iron intake for females aged 11–14 years: NDNS years 7&8. Results are for the total population and by income quintiles. ** One-way Anova significant at the *P* < 0.001 level. * One-way Anova significant at the *P* < 0.05 level. Values are expressed as means ± S.E.M. Supplementary table 3. Percentage contribution of food and food groups to daily zinc intakes females 11–14 years: NDNS years 7&8. Results are for the total population and by income quintiles. ** One-way Anova significant at the *P* < 0.001 level. * One-way Anova significant at the *P* < 0.05 level. Values are expressed as means ± S.E.M. Supplementary table 4. Daily weight of food and food groups consumed by females aged 11–14 years: NDNS years 7&8. Results are for the total population and by income quintiles. **One-way- Anova significant at the *P* < 0.001 level. *One-way Anova significant at the *P* < 0.05 level. Values are expressed as means ± S.E.M. Supplementary table5. Percentage contribution of food and food groups to daily iron intake females 15–18 years. NDNS years 7&8. Results are for the total population and by income quintiles. ** One-way Anova significant at the *P* < 0.001 level. * One-way Anova significant at the *P* < 0.05 level. Values are expressed as means ± S.E.M. Supplementary table 6. Percentage contribution of foods and food groups to daily zinc intake females 15–18 years: NDNS years 7&8. Results are for the total population and by income quintiles. ** One-way Anova significant at the *P* < 0.001 level. * One-way Anova significant at the *P* < 0.05 level. Values are expressed as means ± S.E.M. (DOCX 127 KB)
