# Supplementary material for: Influence of income on diet quality and daily iron and zinc intake: analysis of the National Diet and Nutrition Survey of British females aged 11–14 and 15–18 years
Source: Eur J Nutr. 2022 Sep 23;62(1):499–510. doi: 10.1007/s00394-022-03000-z (PMC9510520; doi:10.1007/s00394-022-03000-z)
Supplement: Supplementary file 1 — Supplementary file1 Supplementary table 1 Plasma ferritin and haemoglobin levels of females in the UK aged 11–14 and 15–18 years and by income quintile. Supplementary table 2. Percentage contribution of food and food groups to daily iron intake for females aged 11–14 years: NDNS years 7&8. Results are for the total population and by income quintiles. ** One-way Anova significant at the P < 0.001 level. * One-way Anova significant at the P < 0.05 level. Values are expressed as means ± S.E.M. Supplementary table 3. Percentage contribution of food and food groups to daily zinc intakes females 11–14 years: NDNS years 7&8. Results are for the total population and by income quintiles. ** One-way Anova significant at the P < 0.001 level. * One-way Anova significant at the P < 0.05 level. Values are expressed as means ± S.E.M. Supplementary table 4. Daily weight of food and food groups consumed by females aged 11–14 years: NDNS years 7&8. Results are for the total population and by income quintiles. **One-way- Anova significant at the P < 0.001 level. *One-way Anova significant at the P < 0.05 level. Values are expressed as means ± S.E.M. Supplementary table5. Percentage contribution of food and food groups to daily iron intake females 15–18 years. NDNS years 7&8. Results are for the total population and by income quintiles. ** One-way Anova significant at the P < 0.001 level. * One-way Anova significant at the P < 0.05 level. Values are expressed as means ± S.E.M. Supplementary table 6. Percentage contribution of foods and food groups to daily zinc intake females 15–18 years: NDNS years 7&8. Results are for the total population and by income quintiles. ** One-way Anova significant at the P < 0.001 level. * One-way Anova significant at the P < 0.05 level. Values are expressed as means ± S.E.M. (DOCX 127 KB) [file 394_2022_3000_MOESM1_ESM.docx]

Influence of income on diet quality and daily iron and zinc intake: Analysis of the National Diet and Nutrition Survey of British females aged 11-14 and 15-18 years.

M. Thomas^1^, L. Coneyworth^1^, J. Pearce^2^ and S. Welham^1^

^1^Division of Nutritional Science, University of Nottingham, Sutton Bonington Campus, Sutton Bonington, Leicestershire LE12 5RD ^2^ Food and Nutrition Subject Group, College of Business, Technology and Engineering, Sheffield Hallam University, Sheffield, S1 1WB

Corresponding author: Simon Welham

Email: simon.welham**@nottingham.ac.uk**

Supplementary table 1. Plasma ferritin and haemoglobin, in females 11-14 and 15-18 years and by income quintile

|  | Total | | | IQ1 | | | IQ2 | | | IQ3 | | | IQ4 | | | IQ5 | | |  |
| --- | --- | --- | --- | --- | --- | --- | --- | --- | --- | --- | --- | --- | --- | --- | --- | --- | --- | --- | --- |
| Age (years) | N | Mean | S.E.M | n | Mean | S.E.M | n | Mean | S.E.M | n | Mean | S.E.M | n | Mean | S.E.M | n | Mean | S.E.M | P value |
| Plasma Ferritin (µg/L) | | | |  |  |  |  |  |  |  |  |  |  |  |  |  |  |  |  |
| 11-14 | 39 | 31.13 | 2.135 | 10 | 34.80 | 4.328 | 3 | 38.33 | 12.875 | 3 | 38.33 | 7.333 | 11 | 25.82 | 3.590 | 7 | 35.14 | 4.183 | 0.366 |
| 15-18 | 39 | 22.77 | 2.806 | 6 | 22.00 | 4.830 | 7 | 19.71 | 5.826 | 5 | 22.20 | 4.164 | 9 | 31.56 | 9.567 | 5 | 22.60 | 5.325 | 0.757 |
| Haemoglobin (g/L) | | | |  |  |  |  |  |  |  |  |  |  |  |  |  |  |  |  |
| 11-14 | 41 | 134.71 | 1.146 | 11 | 135.82 | 2.053 | 3 | 132.67 | 0.882 | 4 | 136.75 | 4.366 | 12 | 132.58 | 2.924 | 6 | 136.00 | 1.807 | 0.786 |
| 15-18 | 42 | 129.74 | 2.002 | 6 | 133.17 | 2.272 | 8 | 128.13 | 1.726 | 4 | 123.75 | 8.290 | 9 | 132.56 | 4.285 | 7 | 134.57 | 3.497 | 0.244 |

**Supplementary table 2.** Percentage contribution of food and food groups to daily iron intake for females aged 11-14 years. NDNS years 7&8.

| **Females 11-14 years** | All | | |  |  |  |  | Income quintile | | | | |  |  |
| --- | --- | --- | --- | --- | --- | --- | --- | --- | --- | --- | --- | --- | --- | --- |
| **Food group** | **n** | **Mean** | **S.E.M** | **1 (n = 23)** | | **2(n = 17)** | | **3 (n = 24)** | | **4 (n = 28)** | | **5 (n = 22)** | | **P value** |
|  |  |  |  | Mean | S.E.M | Mean | S.E.M | Mean | S.E.M | Mean | S.E.M | Mean | S.E.M |  |
| **Cereal and cereal products** | 130 | 52.14 | 1.25 | 51.96 | 3.03 | 55.23 | 3.30 | 49.43 | 2.57 | 53.53 | 3.00 | 50.89 | 2.71 | 0.669 |
| **Of which** |  |  |  |  |  |  |  |  |  |  |  |  |  |  |
| Pasta, rice, pizza, and other cereals | 130 | 12.20 | 1.01 | 16.55 | 3.00 | 12.66 | 3.17 | 9.78 | 2.03 | 13.72 | 2.10 | 7 .40 | 1.56 | 0.074 |
| White bread | 130 | 10.73 | 0.82 | 9.29 | 2.08 | 10.72 | 2.60 | 11.07 | 1.95 | 12.97 | 1.72 | 10.23 | 1.94 | 0.500 |
| Other breakfast cereals | 130 | 8.33 | 1.16 | 6.89 | 2.44 | 12.98 | 4.32 | 4.95 | 2.27 | 11.91 | 3.10 | 3.58 | 1.71 | 0.111 |
| High fibre breakfast cereals | 130 | 7.77 | 1.18 | 6.07 | 2.76 | 7.12 | 2.84 | 11.39 | 3.56 | 3.97 | 1.95 | 10.39 | 2.87 | 0.259 |
| Biscuits | 130 | 4.76 | 0.48 | 3.82 | 0.82 | 2.66 | 0.58 | 4.15 | 1.65 | 5.04 | 0.77 | 7.54 | 1.42 | 0.029 * |
| Buns, cakes, pastries, and fruit pies | 130 | 3.47 | 0.47 | 4.12 | 1.48 | 3.25 | 1.05 | 3.05 | 0.91 | 2.92 | 0.72 | 3.91 | 0.81 | 0.716 |
| Brown, granary, and wheat germ bread | 130 | 2.29 | 0.46 | 2.34 | 1.40 | 2.67 | 1.44 | 2.47 | 1.39 | 1.57 | 0.53 | 3.21 | 1.06 | 0.425 |
| Whole meal Bread | 130 | 1.85 | 0.41 | 1.48 | 0.88 | 2.91 | 1.71 | 2.12 | 0.90 | 1.03 | 0.57 | 3.20 | 1.33 | 0.428 |
| Puddings | 130 | 0.44 | 0.11 | 0 .14 | 0.12 | 0.26 | 0.16 | 0.46 | 0.24 | 0.24 | 0.14 | 1.43 | 0.51 | 0.111 |
| Other Breads | 130 | 0.29 | 0.23 | 1 .28 | 1.28 | 0.00 | 0.00 | 0.00 | 0.00 | 0.15 | 0.15 | 0.00 | 0.00 | 0.637 |
| **Meat and meat products** | 130 | 14.42 | 0.75 | 14.40 | 1.98 | 14.32 | 1.77 | 15.96 | 1.32 | 13.40 | 1.58 | 12.04 | 1.53 | 0.395 |
| **Of which** |  |  |  |  |  |  |  |  |  |  |  |  |  |  |
| Beef and veal dishes | 130 | 3.39 | 0.38 | 3.71 | 1.12 | 4.18 | 0.91 | 3.50 | 1.09 | 2.55 | 0.69 | 3.58 | 0.80 | 0.611 |
| Chicken and turkey dishes | 130 | 3.14 | 0.35 | 2.22 | 0.48 | 3.63 | 0.87 | 3.18 | 0.92 | 3.08 | 0.79 | 3.44 | 0.90 | 0.795 |
| Coated chicken and turkey | 130 | 1.50 | 0.23 | 1.54 | 0.59 | 1.97 | 0.69 | 1.84 | 0.72 | 1.66 | 0.49 | 1.01 | 0.36 | 0.969 |
| Sausages | 130 | 1.34 | 0.21 | 1.07 | 0.51 | 1.48 | 0.85 | 2.41 | 0.62 | 1.29 | 0.36 | 0.70 | 0.30 | 0.190 |
| Bacon and ham | 130 | 1.19 | 0.14 | 1.40 | 0.42 | 0.90 | 0.37 | 1.48 | 0.38 | 1.17 | 0.27 | 0.64 | 0.24 | 0.239 |
| Burgers and kebabs | 130 | 0.83 | 0.19 | 0.91 | 0.55 | 0.75 | 0.41 | 1.19 | 0.60 | 0.87 | 0.36 | 0.66 | 0.29 | 0.976 |
| Lamb and lamb dishes | 130 | 0.78 | 0.26 | 1.15 | 0.76 | 0.00 | 0.00 | 0.43 | 0.24 | 0.52 | 0.42 | 0.28 | 0.20 | 0.609 |
| Pork and pork dishes | 130 | 0.77 | 0.18 | 0.61 | 0.41 | 1.03 | 0.49 | 0.69 | 0.41 | 1.02 | 0.47 | 1.00 | 0.53 | 0.611 |
| Meat pies and pastries | 130 | 0.80 | 0.19 | 0.58 | 0.35 | 0.18 | 0.18 | 1.16 | 0.67 | 0.99 | 0.41 | 0.69 | 0.39 | 0.545 |
| Other meat and meat products | 130 | 0.46 | 0.18 | 1.22 | 0.79 | 0.03 | 0.03 | 0.07 | 0.05 | 0.25 | 0.15 | 0.02 | 0.02 | 0.532 |
| Liver and dishes | 130 | 0.22 | 0.17 | 0.00 | 0.00 | 0.45 | 0.45 | 0.00 | 0.00 | 0.00 | 0.00 | 0.00 | 0.00 | 0.222 |
| **Vegetable and potatoes** | 130 | 12.17 | 0.60 | 14.96 | 1.37 | 10.84 | 1.11 | 12.99 | 1.70 | 11.16 | 1.28 | 11.92 | 1.60 | 0.220 |
| **Of which** |  |  |  |  |  |  |  |  |  |  |  |  |  |  |
| Vegetables (not raw) including vegetable dishes | 130 | 6.14 | 0.47 | 6.27 | 1.08 | 5.77 | 0.99 | 6.39 | 1.28 | 5.67 | 0.95 | 7.09 | 1.33 | 0.943 |
| Chips, fried and roast potatoes and potato products | 130 | 3.07 | 0.33 | 5.23 | 1.23 | 2.98 | 0.62 | 3.17 | 0.63 | 3.18 | 0.74 | 1.32 | 0.28 | 0.064 |
| Potatoes, potato salads and dishes | 130 | 2.09 | 0.25 | 2.67 | 0.69 | 1.44 | 0.28 | 2.53 | 0.75 | 1.24 | 0.39 | 2.58 | 0.59 | 0.240 |
| Salad and other raw vegetables | 130 | 0.86 | 0.11 | 1.00 | 0.28 | 0.65 | 0.20 | 0.85 | 0.31 | 1.07 | 0.25 | 0.93 | 0.23 | 0.534 |

Supplementary table 3. Percentage contribution of food and food groups to daily zinc intakes females 11-14 years: NDNS years 7&8

| Females 11-14 years | All | | | Income quintile | | | | | | | | | | |
| --- | --- | --- | --- | --- | --- | --- | --- | --- | --- | --- | --- | --- | --- | --- |
| Food group | n | Mean | S.E.M | 1 (n = 23) | | 2 (n = 17) | | 3 (n = 24) | | 4 (n = 28) | | 5 (n = 22) | | P value |
|  |  |  |  | Mean | S.E.M | Mean | S.E.M | Mean | S.E.M | Mean | S.E.M | Mean | S.E.M |  |
| Cereal and cereal products | 130 | 31.31 | 0.99 | 31.82 | 2.46 | 31.19 | 2.43 | 30.99 | 1.99 | 32.68 | 2.49 | 31.19 | 2.53 | 0.989 |
| Of which |  |  |  |  |  |  |  |  |  |  |  |  |  |  |
| Pasta, rice, pizza, and other cereals | 130 | 12.31 | 0.88 | 14.46 | 2.13 | 11.80 | 2.27 | 11.57 | 2.13 | 13.82 | 2.09 | 8.96 | 1.90 | 0.242 |
| White bread | 130 | 7.14 | 0.53 | 6.08 | 1.20 | 6.98 | 1.69 | 7.47 | 1.25 | 8.92 | 1.24 | 6.88 | 1.25 | 0.510 |
| Biscuits | 130 | 2.39 | 0.25 | 2.03 | 0.51 | 1.52 | 0.39 | 2.40 | 0.87 | 2.43 | 0.41 | 3.47 | 0.67 | 0.120 |
| High fibre breakfast cereal | 130 | 2.08 | 0.32 | 1.03 | 0.48 | 2.12 | 0.74 | 2.59 | 0.86 | 1.45 | 0.67 | 2.95 | 0.88 | 0.225 |
| Buns, cakes, pastries, and fruit pies | 130 | 2.05 | 0.26 | 2.67 | 0.96 | 1.74 | 0.52 | 1.93 | 0.62 | 1.89 | 0.48 | 2.14 | 0.41 | 0.785 |
| Brown granary and wheat germ bread | 130 | 1.87 | 0.35 | 1.74 | 0.98 | 2.35 | 1.31 | 2.06 | 0.95 | 1.28 | 0.43 | 2.50 | 0.77 | 0.439 |
| Wholemeal bread | 130 | 1.51 | 0.34 | 1.37 | 0.79 | 2.20 | 1.22 | 1.61 | 0.65 | 0.92 | 0.53 | 2.55 | 1.20 | 0.497 |
| Other Breakfast cereals | 130 | 1.20 | 0.19 | 1.07 | 0.48 | 1.86 | 0.69 | 0.86 | 0.42 | 1.66 | 0.45 | 0.42 | 0.24 | 0.145 |
| Puddings | 130 | 0.51 | 0.12 | 0.29 | 0.20 | 0.62 | 0.30 | 0.51 | 0.26 | 0.20 | 0.11 | 1.32 | 0.53 | 0.255 |
| Other breads | 130 | 0.25 | 0.20 | 1.08 | 1.08 | 0.00 | 0.00 | 0.00 | 0.00 | 0.13 | 0.13 | 0.00 | 0.00 | 0.637 |
| Milk and milk products | 130 | 16.24 | 0.90 | 15.38 | 1.72 | 16.33 | 2.60 | 11.84 | 1.68 | 17.73 | 2.32 | 18.45 | 2.11 | 0.172 |
| Of which |  |  |  |  |  |  |  |  |  |  |  |  |  |  |
| Cheese | 130 | 6.36 | 0.65 | 7.13 | 1.56 | 5.02 | 2.15 | 3.92 | 1.19 | 6.92 | 1.52 | 7.51 | 1.34 | 0.082 |
| Semi Skimmed Milk | 130 | 4.99 | 0.60 | 4.88 | 1.31 | 5.40 | 1.84 | 2.91 | 0.90 | 4.45 | 1.49 | 7.53 | 1.60 | 0.126 |
| Whole milk | 130 | 1.70 | 0.40 | 1.59 | 0.76 | 3.89 | 1.62 | 0.87 | 0.44 | 2.25 | 1.26 | 0.30 | 0.26 | 0.205 |
| Yoghurt, fromage frais and other dairy desserts | 130 | 1.63 | 0.22 | 1.21 | 0.55 | 1.20 | 0.57 | 2.16 | 0.70 | 1.31 | 0.44 | 2.30 | 0.46 | 0.074 |
| Other milk and cream | 130 | 0.64 | 0.20 | 0.15 | 0.10 | 0.40 | 0.29 | 0.39 | 0.32 | 1.46 | 0.66 | 0.17 | 0.06 | 0.043* |
| Ice Cream | 130 | 0.48 | 0.09 | 0.42 | 0.16 | 0.32 | 0.18 | 0.38 | 0.18 | 0.69 | 0.25 | 0.45 | 0.24 | 0.571 |
| Skimmed milk | 130 | 0.34 | 0.15 | 0.00 | 0.00 | 0.10 | 0.10 | 1.20 | 0.76 | 0.26 | 0.20 | 0.18 | 0.18 | 0.446 |
| One percent milk | 130 | 0.09 | 0.09 | 0.00 | 0.00 | 0.00 | 0.00 | 0.00 | 0.00 | 0.40 | 0.40 | 0.00 | 0.00 | 0.546 |
| Meat and meat products | 130 | 31.03 | 1.28 | 31.64 | 3.60 | 34.26 | 3.30 | 34.50 | 2.61 | 27.70 | 2.58 | 25.29 | 2.87 | 0.099 |
| Of which |  |  |  |  |  |  |  |  |  |  |  |  |  |  |
| Beef and veal dishes | 130 | 9.21 | 1.01 | 9.43 | 2.85 | 13.41 | 3.02 | 9.59 | 2.72 | 6.78 | 1.65 | 9.56 | 2.33 | 0.515 |
| Chicken, turkey, and Dishes | 130 | 5.99 | 0.64 | 5.26 | 1.14 | 8.30 | 2.38 | 6.33 | 1.92 | 5.79 | 1.28 | 4.79 | 0.73 | 0.824 |
| Bacon and Ham | 130 | 4.16 | 0.46 | 4.53 | 1.26 | 2.63 | 0.98 | 5.28 | 1.29 | 4.41 | 0.96 | 2.35 | 0.82 | 0.378 |
| Burgers and kebabs | 130 | 2.21 | 0.56 | 2.69 | 1.99 | 2.38 | 1.33 | 3.45 | 1.68 | 1.75 | 0.78 | 1.47 | 0.68 | 0.974 |
| Sausages | 130 | 2.09 | 0.31 | 1.66 | 0.77 | 1.84 | 1.11 | 3.58 | 0.90 | 2.15 | 0.58 | 1.24 | 0.50 | 0.191 |
| Pork and Dishes | 130 | 1.94 | 0.40 | 1.66 | 0.96 | 2.89 | 1.24 | 1.85 | 0.95 | 2.13 | 0.91 | 2.43 | 1.14 | 0.660 |
| Lamb and Dishes | 130 | 1.85 | 0.57 | 2.67 | 1.59 | 0.00 | 0.00 | 1.26 | 0.73 | 1.02 | 0.78 | 0.91 | 0.64 | 0.606 |
| Coated chicken and turkey | 130 | 1.83 | 0.28 | 1.51 | 0.51 | 2.42 | 1.11 | 1.86 | 0.67 | 2.18 | 0.69 | 1.43 | 0.47 | 0.976 |
| Meat pies and pastries | 130 | 0.84 | 0.22 | 0.46 | 0.28 | 0.10 | 0.10 | 1.07 | 0.65 | 0.81 | 0.35 | 1.03 | 0.70 | 0.525 |
| Other meat, meat products and dishes | 130 | 0.81 | 0.28 | 1.76 | 1.05 | 0.08 | 0.06 | 0.23 | 0.18 | 0.68 | 0.39 | 0.06 | 0.06 | 0.525 |
| Liver and Dishes | 130 | 0.09 | 0.07 | 0.00 | 0.00 | 0.22 | 0.22 | 0.00 | 0.00 | 0.00 | 0.00 | 0.00 | 0.00 | 0.222 |
| Vegetable and potatoes | 130 | 9.38 | 0.49 | 10.75 | 1.12 | 7.49 | 0.82 | 10.72 | 1.42 | 9.26 | 1.08 | 9.79 | 1.21 | 0.388 |
| Of which | 130 |  |  |  |  |  |  |  |  |  |  |  |  |  |
| Vegetables (not raw) including vegetable dishes | 130 | 4.53 | 0.38 | 3.89 | 0.74 | 3.77 | 0.70 | 5.25 | 1.18 | 4.53 | 0.97 | 5.70 | 0.92 | 0.593 |
| Chips, fried and roast potatoes and potato products | 130 | 2.59 | 0.27 | 4.11 | 0.92 | 2.32 | 0.52 | 2.81 | 0.50 | 3.07 | 0.68 | 1.22 | 0.30 | 0.031* |
| Other potatoes, potato salads and dishes | 130 | 1.66 | 0.20 | 2.16 | 0.53 | 1.01 | 0.20 | 2.12 | 0.60 | 0.95 | 0.27 | 2.11 | 0.44 | 0.222 |
| Salad and other raw vegetables | 130 | 0.59 | 0.07 | 0.59 | 0.22 | 0.39 | 0.12 | 0.54 | 0.16 | 0.71 | 0.15 | 0.76 | 0.19 | 0.487 |

**Supplementary table 4.** Daily weight of food and food groups consumed by females aged 11-14 years. NDNS years 7&8.

| **Females 11-14 years** | **All** | | | **Income quintile** | | | | | | | | | | |
| --- | --- | --- | --- | --- | --- | --- | --- | --- | --- | --- | --- | --- | --- | --- |
| **Food group** | **n** | **Mean** | **S.E.M** | **1 (n = 23)** | | **2 (n = 17)** | | **3 (n = 24)** | | **4 (n = 28)** | | **5 (n = 22)** | | **P value** |
|  |  |  |  | **Mean** | **S.E.M** | **Mean** | **S.E.M** | **Mean** | **S.E.M** | **Mean** | **S.E.M** | **Mean** | **S.E.M** |  |
| **Cereal and Cereal products** | 130 | 237.61 | 7.54 | 217.75 | 17.83 | 268.03 | 15.32 | 186.73 | 14.48 | 254.29 | 14.58 | 260.55 | 18.14 | 0.001* |
| **Of which** |  |  |  |  |  |  |  |  |  |  |  |  |  |  |
| Pasta, rice, pizza, and other cereals | 130 | 104.06 | 6.05 | 113.28 | 16.34 | 119.85 | 19.77 | 71.69 | 8.60 | 115.73 | 12.00 | 87.85 | 12.03 | 0.086 |
| White bread | 130 | 55.47 | 4.15 | 39.40 | 7.44 | 56.76 | 13.13 | 49.44 | 8.39 | 67.53 | 8.60 | 60.63 | 12.01 | 0.299 |
| Buns, cakes, pastries, and fruit pies | 130 | 19.77 | 2.15 | 21.05 | 6.16 | 21.79 | 7.02 | 18.61 | 5.28 | 17.27 | 4.39 | 23.47 | 4.29 | 0.690 |
| Biscuits | 130 | 18.33 | 1.82 | 15.37 | 3.76 | 12.95 | 3.24 | 11.33 | 3.14 | 20.59 | 3.75 | 29.97 | 6.72 | 0.082 |
| Other Breakfast cereals | 130 | 8.80 | 1.40 | 8.29 | 3.88 | 12.43 | 4.25 | 5.85 | 3.39 | 12.31 | 3.51 | 3.48 | 1.61 | 0.109 |
| High fibre breakfast cereal | 130 | 8.77 | 1.37 | 4.25 | 1.99 | 10.76 | 4.20 | 8.88 | 2.88 | 8.38 | 3.81 | 12.38 | 3.56 | 0.285 |
| Brown granary and wheat germ bread | 130 | 7.96 | 1.45 | 5.14 | 2.75 | 9.56 | 5.27 | 7.86 | 3.66 | 5.33 | 1.88 | 13.65 | 4.56 | 0.352 |
| Puddings | 130 | 7.30 | 1.66 | 3.35 | 2.06 | 12.46 | 6.31 | 6.84 | 3.40 | 2.46 | 1.44 | 18.37 | 6.62 | 0.110 |
| Wholemeal bread | 130 | 6.11 | 1.40 | 3.28 | 1.83 | 11.48 | 6.67 | 6.22 | 2.68 | 3.97 | 2.28 | 10.75 | 4.45 | 0.403 |
| Other breads | 130 | 1.04 | 0.79 | 4.35 | 4.35 | 0.00 | 0.00 | 0.00 | 0.00 | 0.71 | 0.71 | 0.00 | 0.00 | 0.637 |
| **Meat and meat products** |  | 104.73 | 4.62 | 89.04 | 11.14 | 115.01 | 11.97 | 100.93 | 9.69 | 99.10 | 8.84 | 107.91 | 13.26 | 0.609 |
| **Of which** |  |  |  |  |  |  |  |  |  |  |  |  |  |  |
| Chicken, turkey, and Dishes | 130 | 34.18 | 2.94 | 25.28 | 5.93 | 45.64 | 10.07 | 29.90 | 6.47 | 31.85 | 6.17 | 39.09 | 7.55 | 0.326 |
| Beef and veal dishes | 130 | 16.59 | 2.29 | 14.29 | 4.14 | 17.11 | 3.69 | 19.32 | 8.50 | 10.50 | 2.97 | 21.27 | 5.63 | 0.467 |
| Coated chicken and turkey | 130 | 12.82 | 1.74 | 10.33 | 3.68 | 15.11 | 5.62 | 10.72 | 3.36 | 15.30 | 4.35 | 12.33 | 3.95 | 0.948 |
| Bacon and Ham | 130 | 12.68 | 1.42 | 11.37 | 3.11 | 11.37 | 4.64 | 13.72 | 3.31 | 13.90 | 3.20 | 7.69 | 2.79 | 0.511 |
| Sausages | 130 | 8.69 | 1.30 | 8.36 | 4.21 | 8.79 | 4.17 | 11.35 | 2.85 | 8.53 | 2.22 | 5.62 | 2.32 | 0.435 |
| Pork and Dishes | 130 | 5.80 | 1.43 | 2.48 | 1.48 | 9.41 | 4.05 | 4.05 | 1.99 | 5.85 | 2.35 | 11.60 | 6.66 | 0.479 |
| Meat pies and pastries | 130 | 4.97 | 1.17 | 4.17 | 2.38 | 1.18 | 1.18 | 5.13 | 3.25 | 6.32 | 2.66 | 5.97 | 3.37 | 0.550 |
| Burgers and kebabs | 130 | 4.22 | 1.04 | 5.94 | 3.88 | 5.34 | 3.27 | 4.64 | 2.48 | 4.22 | 1.70 | 3.05 | 1.63 | 0.976 |
| Lamb and Dishes | 130 | 3.02 | 1.04 | 4.48 | 3.09 | 0.00 | 0.00 | 1.84 | 1.16 | 1.21 | 0.89 | 1.18 | 0.83 | 0.606 |
| Other meat, meat products and dishes | 130 | 1.50 | 0.52 | 2.34 | 1.40 | 0.20 | 0.15 | 0.23 | 0.16 | 1.43 | 0.79 | 0.11 | 0.11 | 0.500 |
| Liver and Dishes | 130 | 0.27 | 0.20 | 0.00 | 0.00 | 0.88 | 0.88 | 0.00 | 0.00 | 0.00 | 0.00 | 0.00 | 0.00 | 0.222 |
| **Vegetable and potatoes** | 130 | 161.36 | 7.18 | 161.83 | 18.06 | 176.58 | 19.94 | 145.03 | 16.06 | 151.62 | 13.17 | 180.54 | 21.43 | 0.757 |
| **Of which** |  |  |  |  |  |  |  |  |  |  |  |  |  |  |
| Vegetables (not raw) including vegetable dishes | 130 | 66.34 | 4.47 | 53.71 | 10.55 | 81.49 | 11.44 | 58.25 | 12.00 | 60.85 | 9.88 | 84.16 | 11.19 | 0.077 |
| Chips, fried and roast potatoes and potato products | 130 | 35.60 | 3.43 | 49.03 | 10.88 | 38.52 | 6.99 | 33.27 | 6.21 | 42.08 | 8.69 | 22.34 | 5.50 | 0.231 |
| Potatoes, potato salads and dishes | 130 | 38.99 | 4.32 | 44.65 | 11.33 | 35.81 | 7.25 | 37.02 | 10.56 | 25.33 | 6.98 | 46.79 | 10.49 | 0.290 |
| Salad and other raw vegetables | 130 | 20.42 | 2.50 | 14.43 | 4.47 | 20.75 | 7.42 | 16.48 | 5.55 | 23.36 | 5.52 | 27.26 | 8.02 | 0.486 |

### **Supplementary table 5.** Percentage contribution of food and food groups to daily iron intake females 15-18 years. NDNS years 7&8.

| Females 15-18 years | All | | |  |  | |  | |  | Income quintile | | | | | | |  |  |
| --- | --- | --- | --- | --- | --- | --- | --- | --- | --- | --- | --- | --- | --- | --- | --- | --- | --- | --- |
| Food group | n | mean | S.E.M | 1 (n = 21) | | | 2 (n = 34) | | | 3 (n = 19) | | | 4 (n = 28) | | | 5 (n = 15) | | P value |
|  |  |  |  | mean | S.E.M | | mean | | S.E.M | mean | | S.E.M | mean | | S.E.M | mean | S.E.M |  |
| Cereal and cereal products | 142 | 45.97 | 1.34 | 51.04 | | 3.91 | 47.05 | 2.70 | | 47.04 | 4.29 | | 45.62 | 2.80 | | 39.32 | 2.45 | 0.256 |
| Of which |  |  |  |  | |  |  |  | |  |  | |  |  | |  |  |  |
| Pasta, rice, pizza, and other cereals | 142 | 10.81 | 0.85 | 16.26 | | 3.09 | 10.11 | 1.33 | | 11.24 | 2.52 | | 8.17 | 1.96 | | 10.70 | 2.37 | 0.184 |
| White bread | 142 | 9.51 | 0.65 | 9.23 | | 1.74 | 10.51 | 1.41 | | 6.39 | 1.15 | | 6.95 | 1.16 | | 10.26 | 1.98 | 0.242 |
| Other breakfast cereals | 142 | 7.25 | 1.11 | 8.02 | | 2.46 | 7.13 | 1.83 | | 8.71 | 3.35 | | 6.38 | 2.45 | | 4.53 | 2.06 | 0.710 |
| High fibre breakfast cereals | 142 | 5.13 | 0.93 | 4.47 | | 2.27 | 7.85 | 2.49 | | 4.39 | 2.03 | | 6.51 | 2.24 | | 3.22 | 1.80 | 0.824 |
| Biscuits | 142 | 4.56 | 0.48 | 4.25 | | 1.25 | 4.14 | 1.04 | | 5.95 | 1.60 | | 5.59 | 1.31 | | 4.70 | 0.90 | 0.564 |
| Buns, cakes, pastries, and fruit pies | 142 | 3.23 | 0.53 | 2.53 | | 0.96 | 4.21 | 1.54 | | 4.89 | 1.55 | | 2.31 | 0.98 | | 2.48 | 1.18 | 0.802 |
| Brown, granary, and wheat germ bread | 142 | 2.88 | 0.47 | 2.52 | | 1.04 | 2.13 | 0.61 | | 1.75 | 0.74 | | 4.75 | 1.79 | | 1.94 | 0.88 | 0.618 |
| Whole meal Bread | 142 | 2.09 | 0.45 | 3.59 | | 1.74 | 0.63 | 0.36 | | 2.32 | 1.46 | | 4.38 | 1.33 | | 1.03 | 0.60 | 0.060 |
| Puddings | 142 | 0.35 | 0.09 | 0.16 | | 0.11 | 0.33 | 0.16 | | 0.32 | 0.26 | | 0.58 | 0.26 | | 0.24 | 0.19 | 0.852 |
| Other Breads | 142 | 0.17 | 0.12 | 0.00 | | 0.00 | 0.00 | 0.00 | | 1.07 | 0.86 | | 0.00 | 0.00 | | 0.22 | 0.22 | 0.092 |
| Meat and meat products | 142 | 17.18 |  | 18.81 | | 2.79 | 17.11 | 2.18 | | 18.30 | 3.02 | | 16.71 | 2.19 | | 11.59 | 2.47 | 0.503 |
| Of which |  |  |  |  | |  |  |  | |  |  | |  |  | |  |  |  |
| Chicken, Turkey and Dishes | 142 | 4.05 | 0.51 | 2.76 | | 0.79 | 5.42 | 1.69 | | 3.54 | 0.94 | | 3.94 | 1.00 | | 2.41 | 0.55 | 0.948 |
| Beef and veal dishes | 142 | 3.62 | 0.47 | 3.13 | | 1.02 | 3.49 | 1.01 | | 4.35 | 1.41 | | 4.11 | 1.09 | | 2.61 | 1.05 | 0.883 |
| Coated chicken and turkey | 142 | 2.48 | 0.35 | 2.49 | | 0.94 | 2.53 | 0.75 | | 2.39 | 1.03 | | 1.65 | 0.51 | | 1.95 | 0.77 | 0.990 |
| Bacon and Ham | 142 | 1.16 | 0.13 | 1.05 | | 0.32 | 0.88 | 0.25 | | 1.03 | 0.30 | | 1.49 | 0.31 | | 0.60 | 0.25 | 0.197 |
| Sausages | 142 | 1.36 | 0.21 | 1.58 | | 0.61 | 1.40 | 0.47 | | 1.21 | 0.51 | | 1.69 | 0.53 | | 0.88 | 0.51 | 0.775 |
| meat pies and pastries | 142 | 1.29 | 0.27 | 2.69 | | 1.06 | 1.03 | 0.37 | | 0.89 | 0.67 | | 0.60 | 0.31 | | 0.28 | 0.28 | 0.155 |
| Burgers and kebabs | 142 | 1.09 | 0.26 | 3.63 | | 1.35 | 0.65 | 0.38 | | 1.52 | 0.70 | | 0.71 | 0.36 | | 0.00 | 0.00 | 0.027 |
| Lamb and Dishes | 142 | 0.92 | 0.28 | 0.70 | | 0.51 | 1.03 | 0.67 | | 1.34 | 0.94 | | 0.66 | 0.49 | | 1.24 | 1.24 | 0.907 |
| Pork and Dishes | 142 | 0.60 | 0.19 | 0.66 | | 0.32 | 0.64 | 0.56 | | 0.22 | 0.11 | | 0.82 | 0.38 | | 0.89 | 0.89 | 0.635 |
| Other meat, meat products and dishes | 142 | 0.61 | 0.24 | 0.12 | | 0.12 | 0.05 | 0.05 | | 1.81 | 1.43 | | 1.05 | 0.61 | | 0.72 | 0.55 | 0.357 |
| Vegetable and potatoes | 142 | 15.02 | 0.77 | 11.09 | | 1.36 | 15.94 | 1.65 | | 11.80 | 1.73 | | 15.01 | 1.99 | | 21.36 | 2.34 | 0.007* |
| Of which |  |  |  |  | |  |  |  | |  |  | |  |  | |  |  |  |
| Vegetables (not raw) including vegetable dishes | 142 | 8.56 | 0.71 | 5.37 | | 1.28 | 9.52 | 1.43 | | 5.33 | 1.28 | | 8.89 | 1.87 | | 14.16 | 2.77 | 0.012* |
| Chips, fried and roast potatoes and potato products | 142 | 3.67 | 0.30 | 3.47 | | 0.96 | 4.28 | 0.76 | | 4.27 | 0.81 | | 2.61 | 0.41 | | 3.52 | 0.78 | 0.515 |
| Potatoes, potato salads and dishes | 142 | 1.83 | 0.20 | 1.49 | | 0.35 | 1.51 | 0.48 | | 1.30 | 0.43 | | 2.46 | 0.51 | | 1.72 | 0.55 | 0.409 |
| Salad and other raw vegetables | 142 | 0.95 | 0.12 | 0.76 | | 0.27 | 0.64 | 0.13 | | 0.90 | 0.29 | | 1.06 | 0.27 | | 1.96 | 0.78 | 0.750 |

**Supplementary table 6.** Percentage contribution of foods and food groups to daily zinc intake females 15-18 years: NDNS years 7&8.

| Females 15-18 years | All | | | Income quintile | | | | | | | | | | |
| --- | --- | --- | --- | --- | --- | --- | --- | --- | --- | --- | --- | --- | --- | --- |
| Food group | n | Mean | S.E.M | 1 (n = 21) | | 2 (n = 34) | | 3 (n = 19) | | 4 (n = 28) | | 5 (n = 15) | | P value |
|  |  |  |  | Mean | S.E.M | Mean | S.E.M | Mean | S.E.M | Mean | S.E.M | Mean | S.E.M |  |
| Cereal and cereal products | 142 | 17.70 | 0.73 | 17.32 | 2.18 | 17.03 | 1.31 | 17.51 | 1.80 | 20.49 | 2.10 | 14.97 | 2.05 | 0.582 |
| Of which |  |  |  |  |  |  |  |  |  |  |  |  |  |  |
| White bread | 142 | 5.93 | 0.42 | 5.24 | 1.01 | 6.96 | 0.94 | 4.32 | 0.79 | 4.35 | 0.78 | 6.29 | 1.69 | 0.312 |
| Brown granary and wheat germ bread | 142 | 2.45 | 0.40 | 2.00 | 0.77 | 2.81 | 1.05 | 4.10 | 1.23 | 1.83 | 0.87 | 1.97 | 0.93 | 0.492 |
| Biscuits | 142 | 2.10 | 0.24 | 2.15 | 0.90 | 1.59 | 0.36 | 2.47 | 0.63 | 2.82 | 0.72 | 2.24 | 0.57 | 0.363 |
| Wholemeal bread | 142 | 1.65 | 0.37 | 3.02 | 1.54 | 0.55 | 0.32 | 1.67 | 0.94 | 3.47 | 1.17 | 0.78 | 0.43 | 0.077 |
| Buns, cakes, pastries, and fruit pies | 142 | 1.54 | 0.24 | 1.08 | 0.39 | 1.39 | 0.48 | 0.91 | 0.34 | 2.25 | 0.76 | 1.12 | 0.52 | 0.789 |
| Pasta, Rice, pizza, and other cereals | 142 | 1.50 | 0.36 | 2.11 | 1.02 | 0.80 | 0.61 | 1.15 | 0.62 | 2.48 | 1.19 | 1.27 | 1.27 | 0.599 |
| High fibre breakfast cereal | 142 | 1.39 | 0.28 | 0.87 | 0.38 | 1.84 | 0.62 | 1.74 | 0.73 | 2.28 | 0.99 | 0.63 | 0.40 | 0.818 |
| Other breads | 142 | 1.13 | 0.24 | 0.87 | 0.36 | 1.09 | 0.32 | 1.16 | 0.51 | 0.97 | 0.42 | 0.68 | 0.33 | 0.834 |
| Other Breakfast cereals | 142 | 0.01 | 0.01 | 0.00 | 0.00 | 0.00 | 0.00 | 0.00 | 0.00 | 0.04 | 0.04 | 0.00 | 0.00 | 0.767 |
| Puddings | 142 | 0.00 | 0.00 | 0.00 | 0.00 | 0.00 | 0.00 | 0.00 | 0.00 | 0.00 | 0.00 | 0.00 | 0.00 | 1.000 |
| Milk and milk products | 142 | 11.04 | 0.74 | 10.16 | 2.03 | 10.14 | 1.43 | 11.43 | 2.33 | 14.10 | 1.63 | 11.87 | 2.60 | 0.295 |
| Of which |  |  |  |  |  |  |  |  |  |  |  |  |  |  |
| Cheese | 142 | 6.73 | 0.68 | 6.77 | 2.00 | 6.83 | 1.29 | 7.30 | 2.15 | 7.17 | 1.46 | 8.23 | 2.72 | 0.917 |
| Other milk and cream | 142 | 1.21 | 0.13 | 0.87 | 0.25 | 1.04 | 0.31 | 1.11 | 0.32 | 1.48 | 0.32 | 1.09 | 0.38 | 0.630 |
| Yoghurt, fromage frais and other dairy desserts | 142 | 1.16 | 0.18 | 1.11 | 0.46 | 0.64 | 0.24 | 0.36 | 0.19 | 2.58 | 0.64 | 1.30 | 0.54 | 0.036 |
| Whole milk | 142 | 0.62 | 0.16 | 0.15 | 0.14 | 0.69 | 0.27 | 0.80 | 0.38 | 0.71 | 0.46 | 0.05 | 0.05 | 0.363 |
| Ice Cream | 142 | 0.62 | 0.13 | 0.63 | 0.31 | 0.81 | 0.38 | 0.14 | 0.10 | 1.00 | 0.35 | 0.42 | 0.19 | 0.500 |
| Semi Skimmed Milk | 142 | 0.51 | 0.15 | 0.63 | 0.55 | 0.03 | 0.03 | 0.67 | 0.40 | 1.11 | 0.48 | 0.56 | 0.37 | 0.086 |
| One percent milk | 142 | 0.13 | 0.10 | 0.00 | 0.00 | 0.00 | 0.00 | 1.01 | 0.69 | 0.00 | 0.00 | 0.00 | 0.00 | 0.034* |
| Skimmed milk | 142 | 0.06 | 0.03 | 0.00 | 0.00 | 0.10 | 0.10 | 0.05 | 0.05 | 0.06 | 0.04 | 0.21 | 0.21 | 0.767 |
| Meat and meat products | 142 | 35.46 | 1.39 | 37.39 | 3.93 | 36.37 | 2.93 | 38.48 | 3.38 | 32.32 | 3.09 | 29.19 | 4.88 | 0.421 |
| Of which |  |  |  |  |  |  |  |  |  |  |  |  |  |  |
| Beef and veal dishes | 142 | 8.80 | 1.07 | 7.75 | 2.40 | 8.96 | 2.35 | 11.28 | 3.28 | 9.45 | 2.35 | 6.46 | 2.76 | 0.860 |
| Chicken, turkey, and dishes | 142 | 6.44 | 0.55 | 4.38 | 0.93 | 7.29 | 1.34 | 5.43 | 0.84 | 5.63 | 1.23 | 5.56 | 1.31 | 0.841 |
| Sausages | 142 | 4.95 | 0.51 | 6.81 | 1.52 | 5.19 | 1.12 | 5.65 | 1.80 | 3.62 | 0.69 | 4.57 | 1.23 | 0.771 |
| Bacon and ham | 142 | 3.77 | 0.40 | 3.35 | 1.05 | 3.18 | 0.78 | 3.07 | 0.76 | 5.02 | 0.97 | 1.80 | 0.75 | 0.205 |
| Coated chicken and turkey | 142 | 3.12 | 0.48 | 3.23 | 1.48 | 3.82 | 1.26 | 2.46 | 0.96 | 1.73 | 0.54 | 2.71 | 1.18 | 0.969 |
| Burgers and kebabs | 142 | 2.38 | 0.58 | 7.91 | 2.85 | 1.45 | 0.91 | 3.29 | 1.62 | 0.99 | 0.56 | 0.00 | 0.00 | 0.026* |
| Meat pies and pastries | 142 | 2.29 | 0.21 | 1.63 | 0.54 | 2.55 | 0.43 | 2.81 | 0.57 | 2.28 | 0.51 | 2.89 | 0.86 | 0.083 |
| Lamb and dishes | 142 | 1.93 | 0.54 | 1.27 | 0.92 | 1.93 | 1.13 | 2.94 | 1.84 | 1.52 | 1.05 | 2.64 | 2.64 | 0.898 |
| Other meat, meat products and dishes | 142 | 1.41 | 0.29 | 0.85 | 0.72 | 1.69 | 0.71 | 1.15 | 0.45 | 1.57 | 0.61 | 2.19 | 1.21 | 0.491 |
| Pork and dishes | 142 | 0.38 | 0.09 | 0.21 | 0.14 | 0.29 | 0.14 | 0.40 | 0.27 | 0.53 | 0.20 | 0.37 | 0.21 | 0.814 |
| Vegetable and potatoes | 142 | 13.16 | 0.90 | 10.85 | 2.52 | 16.30 | 2.03 | 11.01 | 1.45 | 13.22 | 2.31 | 15.68 | 3.08 | 0.185 |
| Of which |  |  |  |  |  |  |  |  |  |  |  |  |  |  |
| Vegetables (not raw) including vegetable dishes | 142 | 7.06 | 0.85 | 5.21 | 2.54 | 8.59 | 1.87 | 4.96 | 1.30 | 7.37 | 2.21 | 11.86 | 3.43 | 0.028* |
| Chips, fried and roast potatoes and potato products | 142 | 3.04 | 0.23 | 2.78 | 0.75 | 3.69 | 0.57 | 3.60 | 0.57 | 2.33 | 0.38 | 2.54 | 0.65 | 0.240 |
| Other potatoes, potato salads and dishes | 142 | 2.00 | 0.29 | 2.34 | 0.83 | 2.21 | 0.70 | 1.61 | 0.61 | 2.87 | 0.78 | 1.15 | 0.61 | 0.692 |
| Salad and other raw vegetables | 142 | 1.06 | 0.31 | 0.53 | 0.32 | 1.81 | 0.90 | 0.84 | 0.37 | 0.66 | 0.29 | 0.13 | 0.11 | 0.407 |
